# Activity of Tivozanib in Non-clear Cell Renal Cell Carcinoma: Subgroup Analysis From a Phase II Randomized Discontinuation Trial

**DOI:** 10.1093/oncolo/oyad132

**Published:** 2023-06-14

**Authors:** Pedro C Barata, Alexander Chehrazi-Raffle, Kimberly D Allman, Aviva Asnis-Alibozek, Vijay Kasturi, Sumanta K Pal

**Affiliations:** Department of Medicine, Case Comprehensive Cancer Center, Seidman Cancer Center, University Hospitals Case Medical Center, Cleveland, OH, USA; Department of Medical Oncology and Therapeutics Research, City of Hope Comprehensive Cancer Center, Duarte, CA, USA; AVEO Oncology, an LG Chem Company, Boston, MA, USA; AVEO Oncology, an LG Chem Company, Boston, MA, USA; AVEO Oncology, an LG Chem Company, Boston, MA, USA; Department of Medical Oncology and Therapeutics Research, City of Hope Comprehensive Cancer Center, Duarte, CA, USA

**Keywords:** tivozanib, non-clear cell, VEGFR-TKI, tyrosine kinase inhibitor, metastatic renal cell carcinoma

## Abstract

**Background:**

Non-clear cell renal cell carcinoma (nccRCC) is a blanket term for a collection of heterogeneous and biologically diverse RCC histologies, including but not limited to papillary, chromophobe, and unclassified subtypes. Tivozanib is a selective vascular endothelial growth factor receptor (VEGFR) tyrosine kinase inhibitor (TKI) that demonstrated activity in RCC with clear cell component. The objective of this analysis was to determine the efficacy of tivozanib in histologically unclassified/mixed RCC.

**Methods:**

We identified patients with nccRCC enrolled in Study 201 (NCT00502307) between October 2007 and July 2008. This was a phase II randomized discontinuation trial of tivozanib in patients with RCC who had no prior VEGFR-targeted treatment. Clinical outcomes including investigator-assessed objective response rate (ORR), disease control rate (DCR, defined by complete response + partial response + stable disease), and progression-free survival (PFS) were examined.

**Results:**

Of the 272 patients enrolled, 46 (16.9%) patients had nccRCC: 11 (4%) papillary, 2 (0.7%) chromophobe, 2 (0.7%) collecting duct, and 31 (11.4%) mixed/unclassified. Of the 46 patients with nccRCC, 38 were continuously treated with tivozanib and the best ORR was 21.1% (confirmed) and 31.6% (confirmed and unconfirmed). The DCR was 73.7% and median PFS was 6.7 months (95% confidence interval, 125-366 days). There were no new safety signals compared to the ITT population. Limitations include the small number of individual nccRCC subtypes and the randomized discontinuation design.

**Conclusion:**

Tivozanib demonstrated activity and a favorable safety profile in patients with nccRCC. These data add to the body of evidence supporting the use of VEGFR-TKI in advanced nccRCC.

## Teaser

Tivozanib is a selective vascular endothelial growth factor receptor tyrosine kinase inhibitor that has demonstrated activity in renal cell carcinoma with clear cell component. This article reports on the efficacy of tivozanib in histologically diverse non-clear cell renal cell carcinoma.

**Implications for Practice**

Treatment options for patients with non-clear cell renal cell carcinoma (nccRCC) are limited. In this study, the authors specifically evaluated the outcomes of patients with nccRCC subtypes who enrolled in a clinical trial investigating the activity of the selective anti-angiogenic therapy tivozanib. It was found that tivozanib was active and well tolerated in patients with different nccRCC subtypes, suggesting tivozanib might be considered a treatment option for this patient population.

## Introduction

Renal cell carcinoma (RCC) has traditionally been divided into 2 categories, clear cell and non-clear cell RCC (nccRCC). The former represents 75%-80% of cases while the latter makes up the remainder.^1^ While clear cell RCC is driven by mutations in *VHL* with consequent increases in vascular endothelial growth factor (VEGF) production, nccRCC represents a diverse array of disease subtypes, each with distinct disease biology.^2^ The most common among these is papillary RCC followed by chromophobe RCC, representing 10%-15% and 4%-5% of RCC cases, respectively.^3,4^ Beyond this, a multitude of other histologies exist (eg, collecting duct carcinoma, medullary RCC, and others), each constituting an even smaller fraction of cases.

Immense progress has been made in the management of advanced clear cell RCC. Harnessing the VEGF dependence of the disease, targeted therapies directed at VEGF and its cognate receptor have been introduced from 2005 onwards.^5,6^ A decade later, immune checkpoint inhibitors (ICIs) were approved. Initially used as second-line therapy, multiple studies have shown the benefit with combination strategies in the front-line setting.^7-9^ As a result, median survival for patients with advanced clear cell RCC has more than tripled since the pretargeted therapy era, at which point cytokine therapy was associated with a median survival of just 1 year.^10^

Outcomes for nccRCC have been more sobering. Studies such as the ESPN and ASPEN, which included multiple non–clear cell histologies, have shown a marginal difference in favor of the VEGFR receptor-tyrosine kinase inhibitor (VEGFR-TKI) sunitinib compared to the mammalian target of rapamycin (mTOR) inhibitor everolimus.^11,12^ Acknowledging that the biology of nccRCC is heterogeneous, one might speculate that a VEGF-directed therapy more potent and specific than sunitinib could elicit greater benefit. Tivozanib, a small molecule inhibitor of VEGFR2 with an IC_50_ of 0.16 nM (as compared to 80 nM for sunitinib), could therefore play a role in this setting.^13,14^ Tivozanib is currently FDA approved for patients with advanced RCC after 2 or more prior systemic treatments.^15^ This approval reflects the TIVO-3 trial, a phase III study in patients with advanced clear cell RCC in which tivozanib showed an improvement in ‑ survival (PFS) and objective response rate (ORR) as compared to the multi-kinase inhibitor sorafenib.^16,17^ In the current manuscript, we turned to a previously reported randomized discontinuation trial of tivozanib in advanced RCC in which a substantial proportion of patients had non–clear clear cell histology to further characterize its activity in this patient subset of unmet need.^18^

## Methods

### Patient Eligibility and Study Design

The study design and methods have been previously reported.^18^ Briefly, patients with unresectable or metastatic RCC were eligible provided they demonstrated a Karnofsky performance status ≥70% and had adequate laboratory and hematologic parameters. Only 1 prior systemic treatment for RCC was permitted so long as it was not a VEGF-directed agent. Patients who had central nervous system involvement or symptomatic disease were excluded, as were patients with poorly controlled hypertension or significant cardiovascular disease (eg, symptomatic left ventricular failure or recent myocardial infarction). The study was approved by local institutional review boards with conduct in accordance with the Declaration of Helsinki.

**Figure 1.**
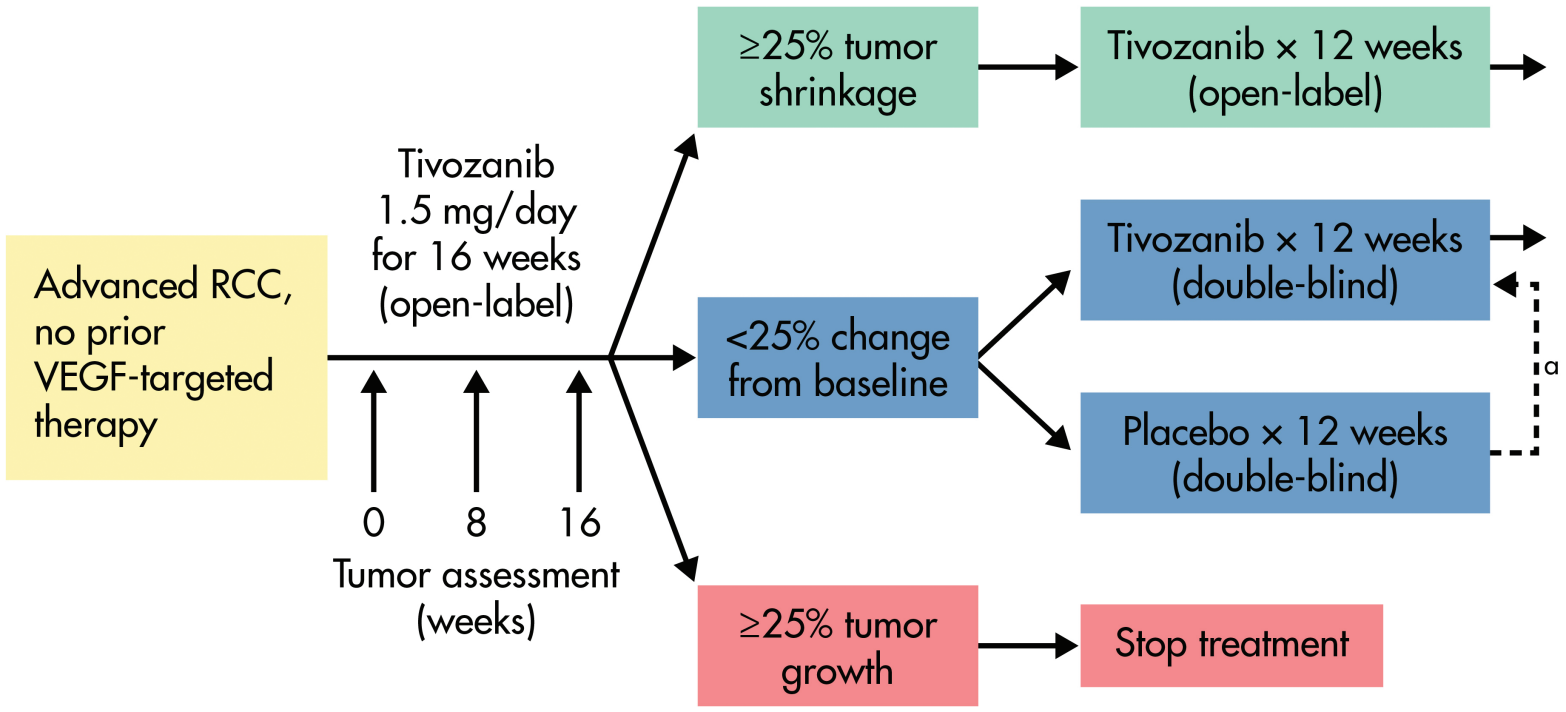
Study design.

The study was structured using a randomized discontinuation scheme (Fig. 1). Enrolled patients received ­open-label tivozanib on a standard schedule of 1.5 mg/day by mouth on a 3-week on, 1-week off cycle. The tivozanib dose studied in this trial is equivalent to the current FDA-approved 1.34 mg capsule. Every 2 cycles, tumor assessments were performed with Response Evaluation Criteria in Solid Tumors (RECIST 1.0) interpretation of response. At 16 weeks, a modified RECIST (mRECIST) criterion was utilized to inform randomization: If patients had ≥25% growth, tivozanib was discontinued. In contrast, those patients with ≥25% shrinkage continued therapy. Those patients with an intermediate response between 25% growth and 25% shrinkage were randomized in a 1:1 fashion (double-blind) to receive either tivozanib or a placebo for an additional 12 weeks. Patients who progressed at any time during this span were unblinded—those receiving placebo could receive subsequent tivozanib while those on tivozanib were removed from the study. At 12 weeks, patients were unblinded and response assessments were conducted. Those individuals with no disease progression and good tolerance were allowed to continue on or resume (placebo patients) open-label tivozanib. After 1 year, treatment with tivozanib may continue at the discretion of the sponsor and the principal investigator.

### Endpoints and Statistical Analysis

The primary endpoint of the study in the overall population and within the randomized subset was based on ORR. As these data have been previously reported, we focused the current analysis on those patients with non-clear cell histology (note: pathology was investigator reported and not centrally confirmed). Investigator-assessed ORR at 16 weeks (mRECIST) in all patients is reported. Additionally, the best confirmed response (which required confirmation on a subsequent scan) and the best unconfirmed response (which included patients for whom a confirmatory scan was not evaluable or who did not demonstrate persistent response beyond one scan) per RECIST 1.0 were assessed in patients continuously treated with tivozanib. These results are presented for the non-clear cell population as a whole and by histologic subtype. PFS was estimated using the Kapan–Meier method for all treated nccRCC patients. For the analysis of PFS, patients who discontinued treatment without progressive disease (PD) or death were censored at their last assessment, and patients who were randomized to receive placebo were censored at the time of randomization.

## Results

### Patient Characteristics

A total of 46 patients were enrolled with a median age of 54 (range: 26-75). Among these 46 patients, the most common histologies were mixed/unclassified (*n* = 31, 67%), papillary (*n* = 11, 24%), chromophobe (*n* = 2, 4%), and collecting duct (*n* = 2, 4%). Patients were primarily male (74%) and White (83%), and a similar proportion of patients with ECOG 0 and 1 performance status were enrolled (41% and 59%, respectively); 50% presented with de novo metastatic disease. Most patients had received no prior therapy (74%), but among patients with prior systemic treatment, interferon monotherapy represented the most commonly received agent (75%; Table 1).

**Table 1.** Patient characteristics.

Demographic	nccRCC, *n* = 46, *n* (%)
Male/female	34 (74)/12 (26)
Age: mean/median Range	55/53.6 years (26-75)
Race: White/Asian	38 (83)/8 (17)
Ethnicity: Non-Hispanic/-Latino	46 (100)
ECOG PS 0/1	19 (41)/27 (59)
Extent of disease at diagnosis:	
* N*+	20 (43)
* M*+	23 (50)
Prior nephrectomy	24 (52)
Prior lines of systemic treatment median (range)	0 (0-1)
Prior systemic treatment	12 (26)
IFN	11
Investigational treatment (± IFN)	3

Abbreviations: nccRCC, non–clear cell renal cell carcinoma; ECOG PS, Eastern Cooperative Oncology Group Performance Status; IFN, interferon.

### Clinical Outcomes

**Figure 2.**
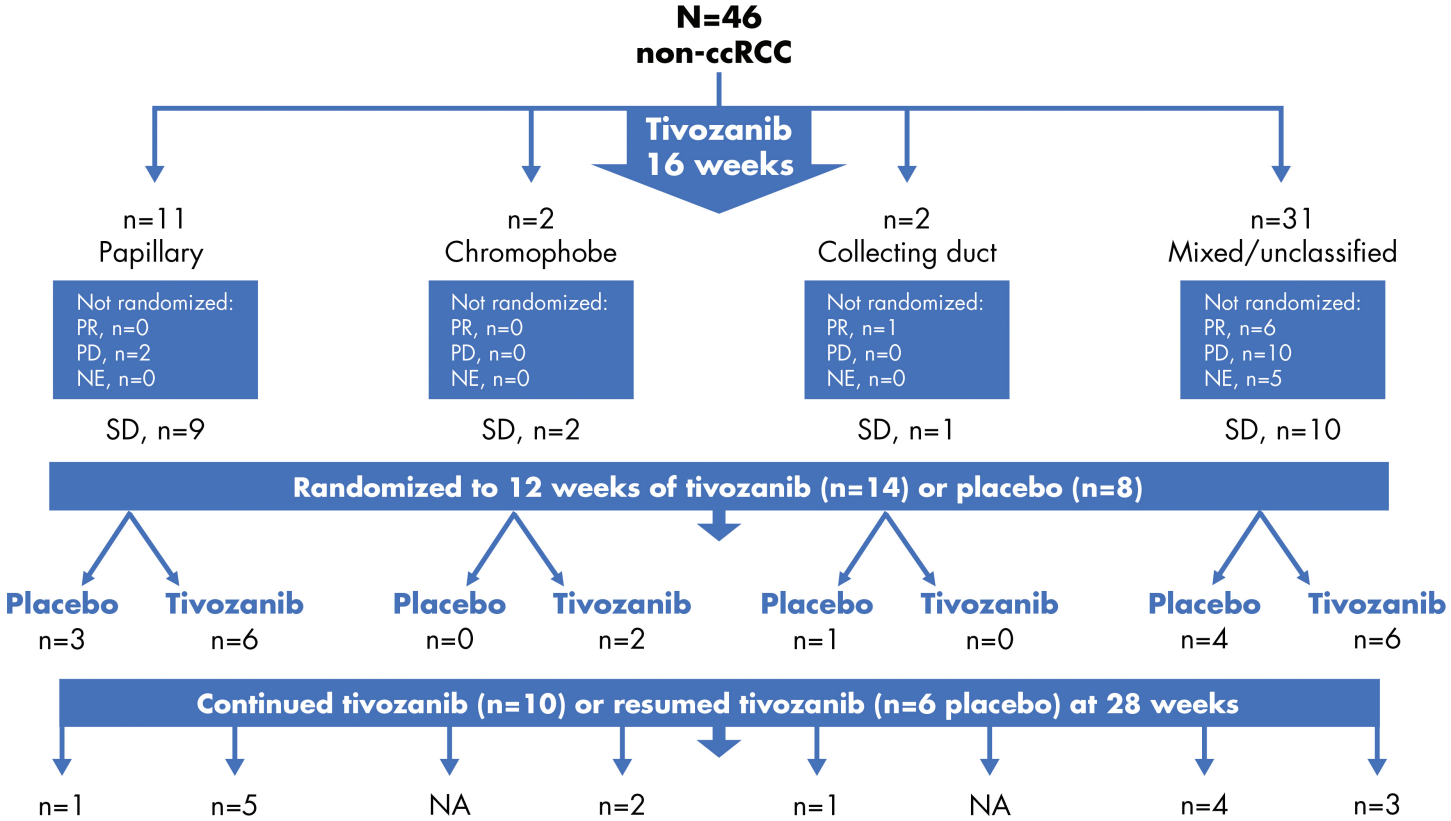
Patient disposition. Abbreviations: NA, not applicable; NE, not evaluable; PD, progressive disease; PR, partial response; SD, stable disease.

At 16 weeks, the ORR (prior to randomization) in all treated patients with nccRCC was 15.2% based on ­investigator-assessed tumor measurements. A total of 7 patients with non-clear cell (15%) achieved tumor shrinkage ≥25% compared with baseline, 22 patients (48%) had tumor measurements within 25% of baseline levels, and 12 patients (26%) demonstrated tumor growth ≥25% at or before Week 16 (Fig. 2). Response assessment was unavailable for 5 patients (11%), all of whom had discontinued treatment prior to Week 12.

Among the 22 patients with <25% tumor growth or shrinkage, 14 patients were randomly assigned to receive tivozanib and 8 patients to placebo. These randomized patients received an average of 9.4 cycles (median 7, range 1-25) of tivozanib or placebo, which corresponds to a mean of 8.4 months (median 6.27) inclusive of the 16 weeks prior to randomization. In total, 38 patients were continuously treated with tivozanib; of the 8 patients randomized to placebo, 6 successfully restarted tivozanib after the 12-week double-blind period.

The best unconfirmed ORR (at any time point) in patients with nccRCC continuously treated with tivozanib was 31.6%, and the best confirmed ORR was 21.1%. When analyzed by nccRCC histologic subset, the best unconfirmed ORR ranged from 22.6% to 100.0% with lower responses among mixed/unclassified histological subtypes (Table 2). Of note, the median time to the best response was 23.2 weeks, and 5 of 12 patients who achieved a partial response (PR) did so at greater than 16 weeks from tivozanib initiation.

**Table 2.** ORR (mRECIST) at Week 16 in all patients and best response (RECIST 1.0) by INV in patients continuously treated with tivozanib.

ORR at Week 16, *n* (%)	All nccRCC, *n* = 46	Papillary, *n* = 11	Chromophobe, *n* = 2	Collecting duct, *n* = 2	Mixed/unclassified, *n* = 31
PR	7 (15.2)	0	0	1 (50)	6 (19.4)
SD	22 (47.8)	9 (81.8)	2 (100)	1 (50)	10 (32.3)
PD	12 (26.1)	2 (18.2)	0	0	10 (32.3)
NE	5 (10.9)	0	0	0	5 (16.1)

Best ORR^*^, *n* (%)	All nccRCC, *n* = 38	Papillary, *n* = 8	Chromophobe, *n* = 2	Collecting duct, *n* = 1	Mixed/unclassified, *n* = 27
PR	12 (31.6)	3 (37.5)	1 (50)	1 (100)	7 (22.6)
SD	16 (42.1)	4 (50)	1 (50)	0	11 (40.7)
PD	6 (15.8)	1 (12.5)	0	0	5 (18.5)
NE	4 (10.5)	0	0	0	4 (14.8)

Best confirmed ORR, *n* (%)	All nccRCC, *n* = 38	Papillary, *n* = 8	Chromophobe, *n* = 2	Collecting duct, *n* = 1	Mixed/unclassified, *n* = 27
PR	8 (21.1)	2 (25)	1 (50)	1 (100)	4 (14.8)
SD	20 (52.6)	5 (62.5)	1 (50)	0	14 (51.9)
PD	6 (15.8)	1 (12.5)	0	0	5 (18.5)
NE	4 (10.5)	0	0	0	4 (14.8)

^*^Includes unconfirmed PR.

Abbreviations: INV, investigator; nccRCC, non-clear cell renal cell carcinoma; NE, not evaluable; ORR, objective response rate; PD, progressive disease; PR, partial response; SD, stable disease.

**Figure 3.**
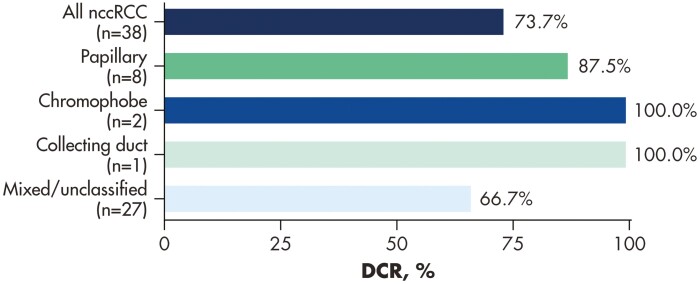
Disease control rate (CR + PR + SD as best response^a^) by nccRCC histology. ^a^Patients continuously treated with tivozanib.

**Figure 4.**
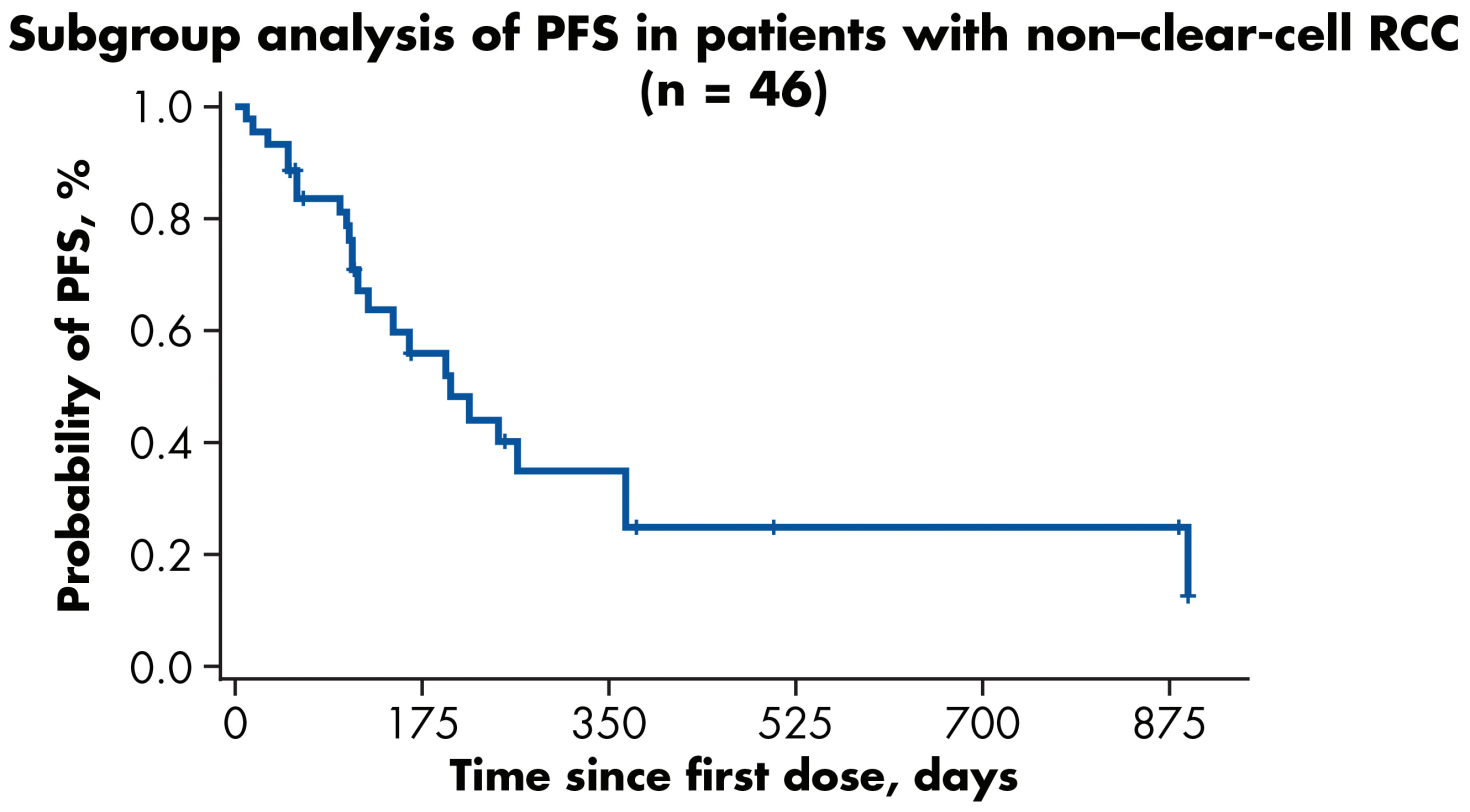
Progression-free survival.

The disease control rate (DCR) was 74% in all patients with nccRCC continuously treated with tivozanib. Figure 3 depicts DCRs by individual nccRCC histological subset. The median PFS for all enrolled nccRCC patients was 6.7 months (95% CI: 3.9-12.0 months; Fig. 4).

### Safety and Tolerability

There were no new safety signals identified with tivozanib. Treatment-related adverse events (TRAEs) occurred in 31 (67%) patients receiving tivozanib (Table 3). The most common TRAEs were hypertension (46%), dysphonia (15%), and asthenia (13%). The most common grade 3/4 adverse events were hypertension (4%), asthenia (2%), and diarrhea (2%). Hyperbilirubinemia and duodenal ulcer hemorrhage were the only Grade 4 TRAEs, each occurring once in a single patient. Across all patients with nccRCC, dose reductions occurred in 4 patients (9%) and 1 TRAE led to treatment discontinuation (duodenal ulcer hemorrhage). No treatment-related deaths were observed.

**Table 3.** Most common (>5% incidence) treatment-related adverse events.

TRAE, *n* (%)	All grade	Grade 1	Grade 2	Grade 3
Hypertension	21 (46)	10 (22)	9 (20)	2 (4)
Dysphonia	7 (15)	7 (15)		
Asthenia	6 (13)	2 (4)	3 (7)	1 (2)
Diarrhea	5 (11)	4 (9)		1 (2)
Fatigue	4 (9)	3 (7)	1 (2)	
Rash	3 (7)	1 (2)	2 (4)	

No grade 5 TRAEs occurred during this study. Two grade 4 TRAEs (duodenal ulcer hemorrhage, hyperbilirubinemia) occurred once in a single patient.

Abbreviation: TRAE, treatment-related adverse event.

## Discussion

This analysis of tivozanib in nccRCC suggests that tivozanib is an effective and well-tolerated therapeutic agent in this patient population. The responses to tivozanib were durable, and patients experienced a median PFS of 6.7 months. TRAEs were in accordance with what was seen in other studies of tivozanib. The adverse event profile is of note because although limited to patients with a clear cell component, the phase III TIVO-3 study completed subsequent to Study 201 demonstrated that tivozanib had better tolerability relative to the comparator VEGFR-TKI sorafenib.^19^ Specifically, tivozanib was associated with less TRAEs, fewer dose modifications, a longer time to onset, and a shorter duration of TRAEs than sorafenib.^19^

Owing to its histologic heterogeneity and relative scarcity, nccRCC has historically been fraught with a paucity of dedicated prospective phase III trials. Consequently, treatment algorithms have largely relied upon retrospective analyses, single-arms trials, and randomized phase II trials. At present, sunitinib is listed as a preferred regimen according to national guidelines.^20^ However, varying activity has been reported with sunitinib in trials including multiple RCC histologies. For instance, in the randomized phase II ASPEN trial (including papillary, chromophobe or unclassified RCC), sunitinib elicited an ORR of 18%.^12^ In the broader catchment of the randomized phase II ESPN trial (which also included these as well as translocation and sarcomatoid RCC), ORR of 9% was observed.^11^

Another preferred agent is cabozantinib, a multi-kinase inhibitor that targets VEGFR 1-3, MET, KIT, AXL, and FLT3.^20,21^ Early retrospective analyses suggested that cabozantinib is biologically active against nccRCC with ORR ranging from 14% to 27%.^22,23^ Recently, a more refined investigational approach has been adopted in which trials have been designed to assess a single histology.^24-26^ The most prominent example of this is the randomized, phase II SWOG 1500 trial, comparing sunitinib to cabozantinib, crizotinib, and savolitinib in patients with papillary RCC.^27^ In this study, cabozantinib emerged as a new standard of care over sunitinib as the former achieved a longer median PFS, higher ORR, and fewer grade 3/4 adverse events. In this analysis, 2 responses were observed among 8 patients with papillary RCC—this 25% ORR in our small subset of patients looks comparable to the 23% ORR associated with cabozantinib in SWOG 1500. It may, therefore, be reasonable to consider future trials of tivozanib in the setting of papillary RCC.

Single-arm phase II studies exclusively including patients with nccRCC have showed encouraging ORR using cabozantinib in combination with either atezolizumab or nivolumab, or bevacizumab with atezolizumab.^28-30^ Furthermore, other TKI/IO combinations such as lenvatinib in combination with pembrolizumab recently reported a confirmed ORR of 48% and a 6-month PFS of 72% in an ongoing phase II trial (NCT04704219) including 149 patients with nccRCC.^31^ Importantly, the higher ORR seen in these studies is somewhat tempered by TRAEs leading to drug discontinuation in 16%-21% of patients. This suggests that tivozanib, with its more tolerable side-effect profile than its VEGFR-TKI counterparts, may be an attractive option for combination therapy. Results from ongoing trials are eagerly awaited to better clarify the role of ICI plus VEGFR-TKI therapy in nccRCC (NCT05043090, NCT04704219, NCT04267120, and NCT04413123).

There are several limitations in this study. Our patient population included small individual RCC subtypes: 31 patients had mixed/unclassified tumors whereas only 11 had papillary RCC. Although tumor heterogeneity is common in nccRCC trials, the high proportion of mixed/unclassified tumors and paucity of papillary RCC deviates from the typical histologic distribution. Further, histologic classification was reported by the investigator, and was not confirmed centrally. Additionally, on account of these data being derived from an older trial, prior and subsequent therapies available to these patients are likely quite different compared to contemporary experiences. In the cited contemporary studies, few patients likely received prior interferon and more patients likely received novel tyrosine kinase inhibitors for refractory disease. Yet, the endpoints reported here such as ORR or PFS and not OS in this front-line study should not be impacted by access to contemporary therapies. Another limitation to our study is the randomized discontinuation design, which required censoring at 16 weeks for patients randomized to placebo. We observed that a good proportion of partial responders achieve their best response after 16 weeks. Therefore, it stands to reason that our PFS and ORR might be underestimated as the patients randomized to the placebo arm may have ultimately derived a more robust response were they to have continued on therapy.

## Conclusions

In summary, tivozanib showed promising clinical activity and was very well tolerated. Additional prospective validation of tivozanib—both as monotherapy and in combination with ICI therapy—in larger cohorts with representation of the different nccRCC subtypes is warranted.

## Data Availability

The data underlying this article will be shared on reasonable request to the corresponding author.
